# Supplementary material for: 3D-ViT-UNet: 3D Vision transformer based Unet-like model for Volumetric Brain Tumor Segmentation
Source: PLOS Digit Health. 2026 Mar 31;5(3):e0001323. doi: 10.1371/journal.pdig.0001323 (PMC13038115; doi:10.1371/journal.pdig.0001323)
Supplement: Attachment — Submitted filename: Reponse to Reviewers.docx [file pdig.0001323.s001.docx]

**Reviewer 1:**

We would like to sincerely thank the reviewers and the editor for their insightful comments and constructive feedback. Their suggestions have significantly contributed to improving the quality and clarity of the manuscript. We deeply appreciate the time and effort they dedicated to reviewing our work. Please note that our responses to the reviewers’ comments are in red in this rebuttal document

**Comment #1. Clarify whether the comparative analysis is conducted under the same experimental configuration across methods (e.g., input resolution, preprocessing). If not, please clearly state the differences and justify the comparison.**

**Response:**
Thank you for raising this point. We have revised the manuscript, and it is to clarify that the comparative analysis is conducted under the same experimental configuration across methods. Specifically, all results reported in our comparison are obtained using the same dataset split, input resolution, modalities, and a consistent preprocessing pipeline (normalization and any resampling/cropping) and inference procedure. These details are clearly specified in the Experimental Setup section.

**Comment #2. What is the specific difference between DIPE and existing dynamic Positional Encoding methods such as Learnable Positional Encoding? What are the advantages?**

**Response:**

In our 3D-ViT-UNet, DIPE (Dynamic Input-Dependent Positional Encoding) differs from Learnable Positional Encoding (LPE) in how the positional signal is generated and how it behaves under resolution changes. In recent ViT-style models, LPE is typically a static learnable embedding associated with patch indices; when the input resolution changes, a common practice is to interpolate the learnable positional embeddings to match the new token grid [1], [2]. Moreover, self-attention is inherently permutation-equivariant, so positional embeddings are required to inject patch order information; learnable positional embeddings are one standard choice [3].

In contrast to conventional fixed position encoding methods, DIPE produces the positional signal from the current input features within each 3D-ViT block using a lightweight depth-wise convolutional generator and residual injection (i.e., $X' = DW-Conv(Resize(X))$and $X_{pos} =Resize(X') + X$, and then all attention projections are computed from this spatially encoded representation, $Q, K, V = Linear(X_{pos})$. This design is aligned with the broader trend toward adaptive positional encodings, where the position encoding is generated conditioned on input tokens or local neighborhoods and can generalize beyond fixed token lengths—e.g., Conditional Positional Encodings (CPE/PEG) explicitly replace fixed/learnable tables with a dynamically generated positional signal [1]. Advantages in our setting are: (1) improved robustness to varying 3D volume without relying on interpolation heuristics used in LPE-based pipelines [1]. (2) stronger spatial consistency by embedding position cues directly into the features that generate (Q,K,V), and (3) clearer mitigation of the transformer’s permutation-equivalence issue because the attention computation becomes explicitly conditioned on spatially encoded token features rather than position-agnostic content alone [3].

[1] Fan, Q., You, Q., Han, X., Liu, Y., Tao, Y., Huang, H., ... & Yang, H. (2024). Vitar: Vision transformer with any resolution. *arXiv preprint arXiv:2403.18361*.

[2] He, K., Gan, C., Li, Z., Rekik, I., Yin, Z., Ji, W., Gao, Y., Wang, Q., Zhang, J., & Shen, D. (2023). Transformers in medical image analysis. Intelligent Medicine, 3 , 59–78.

[3] Das, B. K., Zhao, G., Islam, S., Re, T. J., Comaniciu, D., Gibson, E., & Maier, A. (2024). Co-ordinate-based positional embedding that captures resolution to enhance transformer’s performance in medical image analysis. *Scientific Reports*, *14*(1), 9380.

**Comment #3. Based on what content is the dilation factor selected for 3D-DW-MSA?**

**Response:**

In 3D-DW-MSA, the dilation factor corresponds to the dilation interval used to sample patches when forming each 3D dilated-window. We select this factor deterministically from the current stage’s feature-map patch-grid size *(D, W, H)* and the local 3D-window (unit) size *(*$p_{D},p_{W},p_{H}$*)* using:

$$r_{D}=\frac{D}{p_{D}},r_{H}=\frac{H}{p_{H}},r_{W}=\frac{W}{p_{W}}.$$

This choice ensures that 3D-DW-MSA expands the receptive field from local to global range by sampling one patch every $r$-th position along each axis, while keeping the number of attended tokens per window constant $\left( N = p_{D}p_{H}p_{W} \right)$, thereby maintaining comparable computational cost. For example, as illustrated in Fig. 2, for a feature map of size $(6\times6\times6$) and a window of size $(3\times3\times3$), we obtain $r_{D}=r_{H}=r_{W}=2$, meaning patches are selected at interval 2 to cover the global volume without increasing tokens. Also, this design is consistent with the receptive-field expansion principle of dilated convolutions ref: dilated convolutions [1].

[1] Amer, A., Ye, X., Zolgharni, M., & Janan, F. (2020). Resdunet: Residual dilated unet for left ventricle segmentation from echocardiographic images. In 2020 42nd annual international conference of the IEEE Engineering in Medicine & Biology Society (EMBC) (pp. 2019–2022). IEEE.

**Comment #4. The terms over-segmentation and under-segmentation for brain tumor sub-region segmentation (WT/TC/ET) have been used in the paper. Cite key studies that using these terms and briefly state what each means for the predicted masks.**

**Response:**

In brain tumor segmentation, over-segmentation and under-segmentation describe spatial inaccuracies where the predicted mask deviates from the ground truth. Over-segmentation occurs when a model classifies healthy tissue or background pixels as tumorous, leading to an overestimation of the sub-region volume and an increase in false positives. Conversely, under-segmentation occurs when the model fails to capture the full extent of the tumor, resulting in a predicted mask that is smaller than the actual ground truth, thereby increasing false negatives. These terms are critical for evaluating model precision in the Whole Tumor (WT), Tumor Core (TC), and Enhancing Tumor (ET) sub-regions, as they differentiate between types of errors that a standard Dice score might obscure.

Recent literature highlights these phenomena as central to improving diagnostic accuracy. Early foundational work by [1] investigated the crucial trade-off between over- and under-segmentation, defining an optimal performance value at the cross-point between the two error types. Building on this, the study on [2] formally introduced the Over-segmentation index (OS) and Under-segmentation index (US) to provide a more granular evaluation of boundary precision than traditional metrics. Additionally, research published in [3] and CNN-Based Brain Tumor Approaches utilizes these terms to analyze the performance of U-Net architectures, specifically noting that multi-scale features are essential to mitigate under-segmentation in complex, small regions like the ET. Furthermore, balancing both error types is vital for clinical treatment planning where tumor diameter and area measurements are paramount is emphasized by [4].

[1] Troya-Galvis, A., Gançarski, P., Passat, N., & Berti-Equille, L. (2015). Unsupervised quantification of under-and over-segmentation for object-based remote sensing image analysis. *IEEE Journal of Selected Topics in Applied Earth Observations and Remote Sensing*, *8*(5), 1936-1945.

[2] JS, N. (2025). Brain tumor segmentation using multi-scale attention U-Net with EfficientNetB4 encoder for enhanced MRI analysis. *Scientific Reports*, *15*(1), 1-20.

[3] Xu, Y., Quan, R., Xu, W., Huang, Y., Chen, X., & Liu, F. (2024). Advances in medical image segmentation: A comprehensive review of traditional, deep learning and hybrid approaches. *Bioengineering*, *11*(10), 1034.

[4] Yuan, J. (2024). Brain tumor image segmentation method using hybrid attention module and improved mask RCNN. *Scientific Reports*, *14*(1), 20615.

**Comment #5. What role does the DIPE play in 3D-ViT-UNet, and how does it solve the permutation equivalence problem of the transformers?**

**Response:**

In 3D-ViT-UNet, DIPE (Dynamic Input-Dependent Positional Encoding) is introduced to explicitly inject 3D spatial location information into patch tokens before self-attention. DIPE generates a position-aware feature map via a lightweight depth-wise 3D convolution and residual injection, $X' = DW-Conv(Resize(X))$and $X_{pos} =Resize(X') + X$, and then all attention projections are computed from this spatially encoded representation, $Q, K, V = Linear(X_{pos})$. Hence, DIPE acts as a spatial identity carrier for tokens throughout the encoder’s 3D-ViT blocks [1].

This design directly targets the permutation-equivalence (permutation-equivariance) limitation of vanilla transformers [2]: without positional information, self-attention primarily models content similarity and is largely insensitive to the original 3D ordering of tokens, meaning different permutations of the same patch set can yield equivalent attention behavior. For voxel-wise 3D segmentation, this is problematic because the network must learn not only “what” features exist, but also “where” they belong anatomically. By embedding learned positional cues into $X_{pos}$, DIPE makes attention responses depend on both content and spatial position; therefore, permuting tokens changes $X_{pos}$and consequently changes *(Q,K,V)* and the attention output. As a result, DIPE preserves absolute and relative 3D spatial relationships and enables spatially coherent tumor subregion segmentation.

[1] He, K., Gan, C., Li, Z., Rekik, I., Yin, Z., Ji, W., Gao, Y., Wang, Q., Zhang, J., & Shen, D. (2023). Transformers in medical image analysis. Intelligent Medicine, 3 , 59–78.

[2] Das, B. K., Zhao, G., Islam, S., Re, T. J., Comaniciu, D., Gibson, E., & Maier, A. (2024). Co-ordinate-based positional embedding that captures resolution to enhance transformer’s performance in medical image analysis. *Scientific Reports*, *14*(1), 9380.

**Comment #6. The manuscript refers to fine-grained details and over-smoothing. Provide appropriate citations and a brief clarification to substantiate these claims and to explain their relevance to 3D brain tumor segmentation.**

**Response:**

Thank you for the comment. In the context 3D brain tumor segmentation, fine-grained details and over-smoothing refer to the preservation versus the loss of critical morphological and textural information during feature extraction. Fine-grained details encompass high-frequency components, such as sharp tumor boundaries and the irregular projections of the enhancing tumor (ET). Preserving these is vital because brain tumors are highly heterogeneous; losing small-scale spatial information leads to clinical misidentification of margins. Conversely, over-smoothing is a common artifact in deep learning models—often caused by heavy pooling or excessive regularization—resulting in "blurry" masks that fail to capture intricate structures and lead to a significant loss of semantic content in feature maps.

Recent literature emphasizes that mitigating over-smoothing is essential for reliable segmentation. The SwinCLNet Framework in [1] highlights the necessity of detailed extraction blocks to maintain high-precision segmentation in all sub-regions (WT/TC/ET) and avoid the over-smoothing typical of standard convolutional layers. Similarly, research in [2], demonstrates that focusing on fine-grained geometry near boundaries achieves superior structure continuity. Studies such as the Deep Fusion Model in [2] and Hybrid Attention Analysis in [3] further substantiate these claims, noting that models must preserve subtle gradients to prevent the "loss of semantic content" and idiosyncratic intensity distributions peculiar to complex brain tumors.

[1] Jin, S., Noh, Y., Moon, H., Lee, M., & Noh, W. (2025). SwinCLNet: a robust framework for brain tumor segmentation via shifted window attention and cross-scale fusion. *Scientific Reports*.

[2] Islam, N., Bhuiyan, M. I., Raya, J. T., Kamarudin, N. S., Hasib, K. M., Mridha, M. F., & Farid, D. M. (2024). Deep fusion model for brain tumor classification using fine-grained gradient preservation. *arXiv preprint arXiv:2406.19690*.

[3] Shoaib, M. R., Zhao, J., Emara, H. M., Mubarak, A. S., Omer, O. A., Abd El-Samie, F. E., & Esmaiel, H. (2025). Improving brain tumor classification: An approach integrating pre-trained CNN models and machine learning algorithms. *Heliyon*, *11*(10).

**Comment #7. What are the advantages of pure 3D ViT architectures over hybrid architectures such as Swin-Unet?**

**Response:**
A pure 3D ViT encoder–decoder (as in our 3D-ViT-UNet) offers several advantages over hybrid architectures that mix convolutional components with transformer components. First, it provides a unified, end-to-end token representation across all stages: features are consistently modeled as 3D patch tokens and processed by attention-based blocks. It reduces the representation mismatch that can occur when CNN feature pyramids are later fused with transformer tokens. This is especially beneficial in volumetric segmentation, where maintaining global 3D spatial consistency across depth–height–width is critical [1], [2].

Second, a pure 3D ViT can explicitly control the local–global context trade-off inside the same attention framework. In our case, 3D-W-MSA captures strong local context efficiently, while 3D-DW-MSA expands the receptive field to long-range/global context at controlled cost (by keeping the attended token count per window fixed). This allows to model long-range dependencies and cross-region tumor context without relying on separate CNN inductive biases.

Finally, applying a positional strategy like DIPE becomes more coherent in a pure transformer pipeline. Positional information is injected in a consistent manner into the attention computation across transformer blocks (rather than being partially implied by convolutions and partially learned by attention). It helps preserve spatial identity for voxel-wise segmentation.

[1] Liu, X., Gao, P., Yu, T., Wang, F., & Yuan, R.-Y. (2025). Cswin-unet: Transformer unet with cross-shaped windows for medical image segmentation. Information Fusion, 113 , 102634.

[2] Jiang, Y., Zhang, Y., Lin, X., Dong, J., Cheng, T., & Liang, J. (2022). Swinbts: A method for 3d multimodal brain tumor segmentation using swin transformer. Brain sciences, 12 , 797.

**Reviewer 2:**

**Comment #1. In Table 4, what is the size of the training and validation data sets for the given model? Is the comparison with existing methods based on the same protocol?**

**Response:**

We used the BraTS 2020 training set (369 labeled cases) for both training and validation by applying a 75%-25% split for training and validation respectively, resulting in 277 training cases and 92 validation cases. The official BraTS 2020 validation set (125 cases) was not used for supervised training/validation because its ground-truth labels are not released; the challenge organizers [1].

This protocol is consistent with common practice in recent BraTS 2020 studies. For example, [1] explicitly note that the BraTS 2020 validation set does not contain ground truth and therefore perform internal cross-validation on the 369-case training set for validation [2]. Similarly, only the BraTS training set is used by [3], because it is the only subset with publicly available ground truths.

[1] Bakas, P. S. (2020). Multimodal brain tumor seg-mentation challenge 2020: Data. *Perelman School of Medicine, University of Pennsylvania*.

[2] Ma, B., Sun, Q., Ma, Z., Li, B., Cao, Q., Wang, Y., & Yu, G. (2024). DTASUnet: a local and global dual transformer with the attention supervision U-network for brain tumor segmentation. *Scientific Reports*, *14*(1), 28379.

[3] Yoo, J. J., Namdar, K., Wagner, M. W., Yeom, K. W., Nobre, L. F., Tabori, U., ... & Khalvati, F. (2025). Generative AI for weakly supervised segmentation and downstream classification of brain tumors on MR images. *Scientific Reports*, *15*(1), 22160.

**Comment #2. DIPE is employed in the encoder to encode patch positions. Please discuss whether positional encoding is also necessary in the decoder for reconstructing spatial correspondence in transformer-based decoding blocks, and justify the current design choice.**

**Response:**

In our 3D-ViT-UNet implementation DIPE is used in both the encoder and the decoder, because it is integrated within each 3D-ViT block as a positional module. Specifically, DIPE is applied before the attention blocks in the encoder and is likewise applied in the corresponding decoder stages, preserving positional identity during transformer-based decoding as well.

From a design perspective, an explicit positional signal remains beneficial in decoder transformer blocks whenever attention is used, since attention is otherwise weakly constrained by spatial order. Keeping DIPE inside the block provides spatially grounded decoding at each resolution while remaining compatible with varying 3D sizes because the positional signal is computed from the current feature map rather than a fixed index table. This is consistent with recent segmentation-transformer directions that emphasize dynamic positional modeling to improve spatial detail learning such dynamic/relative positional strategies in [1] and [2].

[1] Sofiani, I. R., Suyono, H., Yudaningtyas, E., & Utaminingrum, F. (2025). Multi-level spatio-relational segformer (MLSRS-SegFormer): A novel vision transformer with adaptive spatial induction and dynamic positional encoding. *MethodsX*, 103693.

[2] Huang, Z., Cheng, S., & Wang, L. (2024). Medical image segmentation based on dynamic positioning and region-aware attention. *Pattern Recognition*, *151*, 110375.

**Comment #3. Since transformer related work has been widely explored in brain tumor segmentation, what are the advantages of the given model compared with existing works?**

**Response:**

We agree that transformer-based segmentation has been widely studied UNETR [1], TransUNet [2]. TransBTS [3], TransFuse [4] and more recent MISSFormer [5], Swin-Unet [6], TransDeepLab [7], UNETR++, TMA-TransBTS [8]. However, many existing approaches still face three recurring limitations for volumetric brain tumor segmentation: (i) several methods process 3D MRIs in a 2D slice-wise manner, which can lose true 3D spatial contextual information; (ii) vanilla self-attention can suffer from loss of positional order (permutation-equivariant behavior) if positional cues are insufficient or fixed, which is harmful for spatially dependent voxel-wise segmentation; and (iii) global 3D attention is often computationally expensive, while some efficiency-oriented designs may still struggle to achieve sufficient global information fusion on large 3D volumes.

Our 3D-ViT-UNet is designed specifically to address these points with a unified, end-to-end 3D transformer U-shape pipeline. (1) It processes tokenized 3D volumetric patches directly, preserving 3D spatial dependencies throughout the encoder–decoder. (2) It combines 3D-W-MSA (efficient local 3D window attention) with 3D-DW-MSA (dilated-window attention) to capture both local and global context; importantly, 3D-DW-MSA expands the receptive field without increasing the number of attended patches, maintaining computational efficiency compared to dense global attention. (3) We integrate DIPE (depth-wise convolution based dynamic input-dependent positional encoding) to encode absolute and relative patch positions, preserving the spatial order of patches and mitigating the transformer’s permutation-equivalence issue, thereby improving spatial coherence in voxel-wise segmentation. Together with the U-shaped skip-connected design, these components provide a practical accuracy–efficiency trade-off and more spatially consistent volumetric segmentation compared with prior transformer baselines under comparable settings.

[1] Hatamizadeh, A., Tang, Y., Nath, V., Yang, D., Myronenko, A., Landman, B., Roth, H. R., & Xu, D. (2022a). Unetr: Transformers for 3d medical image segmentation. In Proceedings of the IEEE/CVF winter conference on applications of computer vision (pp. 574–584).

[2] Chen, J., Lu, Y., Yu, Q., Luo, X., Adeli, E., Wang, Y., Lu, L., Yuille, A. L., & Zhou, Y. (2021). Transunet: Transformers make strong encoders for medical image segmentation. arXiv preprint arXiv:2102.04306 , .

[3] Wenxuan, W., Chen, C., Meng, D., Hong, Y., Sen, Z., & Jiangyun, L. (2021). Transbts: Multimodal brain tumor segmentation using transformer. In International Conference on Medical Image Computing and Computer-Assisted Intervention, Springer (pp. 109–119).

[4] Zhang, Y., Liu, H., & Hu, Q. (2021). Transfuse: Fusing transformers and cnns for medical image segmentation. In Medical image computing and computer assisted intervention–MICCAI 2021: 24th international conference, Strasbourg, France, September 27–October 1, 2021, proceedings, Part I 24 (pp. 14–24). Springer.

[5] Huang, X., Deng, Z., Li, D., Yuan, X., & Fu, Y. (2022). Missformer: An effective transformer for 2d medical image segmentation. IEEE transactions on medical imaging, 42 , 1484–1494.

[6] Cao, H.,Wang, Y., Chen, J., Jiang, D., Zhang, X., Tian, Q., &Wang, M. (2022). Swin-unet: Unet-like pure transformer for medical image segmentation. In European conference on computer vision (pp. 205–218). Springer.

[7] Azad, R., Heidari, M., Shariatnia, M., Aghdam, E. K., Karimijafarbigloo, S., Adeli, E., & Merhof, D. (2022). Transdeeplab: Convolution-free transformer-based deeplab v3+ for medical image segmentation. In International Workshop on PRedictive Intelligence In MEdicine (pp. 91–102). Springer.

[8] Huang, Y., Chen, L., & Zhou, C. (2025). Multi-modal brain tumor segmentation via 3d multi-scale self-attention and cross-attention. arXiv preprint arXiv:2504.09088 , .

**Comment #4. How does DIPE adapt to varying 3D input sizes (e.g., different volume dimensions or patch grids.**

**Response:**

DIPE is architecturally flexible because it does not rely on a fixed-length positional embedding table tied to a specific patch grid. Instead, DIPE generates the positional signal **from the current input feature map** using a lightweight depth-wise convolution and residual injection, i.e., $X^{'}=\text{DW-Conv}(\text{Resize}(X))$and $X_{\text{pos}}=\text{Resize}(X^{'})+X$. Since the depth-wise convolution is applied over the spatial dimensions and the positional features are computed directly from the input tensor, DIPE naturally supports different 3D volume dimensions and patch-grid resolutions; when the input size changes, the DIPE output changes accordingly while preserving spatial correspondence. In practice, DIPE operates on the stage-specific feature map produced by the encoder, so no separate learned position table or re-parameterization is needed, and any resizing is purely to match the tensor shapes required by the attention block rather than to interpolate fixed positional embeddings.

**Comment #5. In the related work section, the authors should add the subheadings to make the description clearer.**

**Response:**

This change has been incorporated in the revised manuscript.

**Comment #6. Provide a clear justification for using two 3D-ViT blocks in the bottleneck. In particular, explain what representational or performance benefits this design offers compared to using a single block or alternative bottleneck configurations.**

**Response:**

In our 3D-ViT-UNet, the bottleneck utilizes two consecutive 3D-ViT blocks to maximize representational capacity at the highest level of abstraction. While a single block often fails to capture complex multi-scale interactions, this dual-block design allows the first block to aggregate global spatial relationships while the second refines these features into a cleaner, more discriminative representation for the decoder. As noted in the **[1]**, stacking transformer blocks at this stage significantly reduces over-smoothing by enabling complex non-linear mappings that single-block or CNN-based bottlenecks cannot achieve.

This configuration provides a superior accuracy–efficiency trade-off, as self-attention costs are lowest on the reduced bottleneck grid. While adding more than two blocks risks overfitting on BraTS datasets and yields diminishing returns, the dual-block approach ensures sufficient depth to distinguish between tumor core and periphery.

[1] Jin, S., Noh, Y., Moon, H., Lee, M., & Noh, W. (2025). SwinCLNet: a robust framework for brain tumor segmentation via shifted window attention and cross-scale fusion. *Scientific Reports*.
